# Disruption of MEF2C signaling and loss of sarcomeric and mitochondrial integrity in cancer-induced skeletal muscle wasting

**DOI:** 10.18632/aging.100436

**Published:** 2012-02-21

**Authors:** Angie M. Y. Shum, Theodore Mahendradatta, Ryland J. Taylor, Arran B. Painter, Melissa M. Moore, Maria Tsoli, Timothy C. Tan, Stephen J. Clarke, Graham R. Robertson, Patsie Polly

**Affiliations:** ^1^ Inflammation and Infection Research Centre, School of Medical Sciences, Faculty of Medicine, University of New South Wales, Sydney, NSW, 2052, Australia; ^2^ Department of Pathology, School of Medical Sciences, Faculty of Medicine, University of New South Wales, Sydney, NSW, 2052, Australia; ^3^ Cancer Pharmacology Unit, ANZAC Research Institute, University of Sydney at Concord Repatriation and General Hospital, Sydney, NSW, 2139, Australia

**Keywords:** Cancer cachexia, IL-6, MEF2C, Myofibril loss, Mitochondria, Colon 26 (C26) carcinoma

## Abstract

Cancer cachexia is a highly debilitating paraneoplastic disease observed in more than 50% of patients with advanced cancers and directly contributes to 20% of cancer deaths. Loss of skeletal muscle is a defining characteristic of patients with cancer cachexia and is associated with poor survival. The present study reveals the involvement of a myogenic transcription factor Myocyte Enhancer Factor (MEF) 2C in cancer-induced skeletal muscle wasting. Increased skeletal muscle mRNA expression of *Suppressor of Cytokine Signaling* (*Socs*) 3 and the *IL-6 receptor* indicative of active IL-6 signaling was seen in skeletal muscle of mice bearing the Colon 26 (C26) carcinoma. Loss of skeletal muscle structural integrity and distorted mitochondria were also observed using electron microscopy. Gene and protein expression of MEF2C was significantly downregulated in skeletal muscle from C26-bearing mice. MEF2C gene targets *myozenin* and *myoglobin* as well as *myokinase* were also altered during cachexia, suggesting dysregulated oxygen transport capacity and ATP regeneration in addition to distorted structural integrity. In addition, reduced expression of calcineurin was observed which suggested a potential pathway of MEF2C dysregulation. Together, these effects may limit sarcomeric contractile ability and also predispose skeletal muscle to structural instability; associated with muscle wasting and fatigue in cachexia.

## INTRODUCTION

Cachexia is a hypermetabolic wasting syndrome involving the progressive depletion of adipose tissue and skeletal muscle mass, irrespective of nutritional intake. It has the highest incidence in patients with gastrointestinal and pancreatic cancers (83-87%) [1]. At least 20% of cancer deaths are directly caused by cachexia [1]. Cachexia is associated with severe weight loss, muscle wasting, weakness and fatigue and causes psychosocial distress and dependency upon others; significantly impairing quality of life [2]. Importantly, reversal of weight loss has been shown to be associated with increased longevity in tumor-bearing (TB) mice [3]. Therefore, a better understanding of the molecular process underlying skeletal muscle wasting due to cancer cachexia is critical in designing intervention strategies; beneficing both the quality and longevity of cachectic patients.

Inappropriate chronic stimulation of downstream signaling molecules by constant influx of tumor and host-derived cytokines affects normal homeostasis and energy metabolism, resulting in altered muscle respiration, muscle fatigue and degradation. Raised levels of Interleukin (IL)-1β, IL-6, Tumor Necrosis Factor-alpha (TNF-α) are implicated as tumor-derived factors in cachexia [1]. TNF-α and Interferon-gamma (IFN-γ) have been shown to be capable of downregulating muscle-specific gene products and activating Nuclear Factor-kappa B (NF-κB) resulting in impaired myogenesis [4]. Additionally, there is substantial evidence implicating a role of IL-6 in the pathogenesis of cancer cachexia [5]. Development of cachexia in pre-clinical mouse models and clinical data correlate with increased circulating IL-6 [6-11]. Blockade of IL-6 action has significantly reversed skeletal muscle wasting in rodent models [11-14]. Moreover, IL-6 alone has been shown to cause skeletal myofiber changes; both structural and metabolic [15, 16]. Therefore, a complex network of cytokine signaling is involved in the pathogenesis of cancer cachexia and cytokines such as IL-6, may play a dominant role.

IL-6 is a pleiotropic cytokine which has both pro- and anti-inflammatory properties [17]. IL-6 mediates its action via IL-6 receptor (IL-6RA) which hetero-dimerizes with the gp130 partner [18]. The JAK-STAT signaling pathway is then activated and promotes the transcription of Suppressor of Cytokine Signaling (SOCS) 3 as a negative feedback mechanism [18]. SOCS3 has been shown to interact with molecules in metabolic pathways and modulate their signaling [19]. Therefore, this raises the question of whether signaling involving SOCS3 may crosstalk with myogenic pathways under chronic IL-6 stimulation during cancer cachexia.

Myocyte Enhancer Factor (MEF) 2C is a myogenic transcription factor that plays a critical role in skeletal muscle development and differentiation. It regulates genes encoding proteins involved in maintaining sarcomeric integrity and function, such as M-line and Z-line proteins [20]. The role of MEF2C in postnatal muscle maintenance has been highlighted in a skeletal muscle specific *Mef2c* knockout mouse model, whereby sarcomeres appeared to be disorganized and fragmented [20]. MEF2 also regulates genes with diverse functions in skeletal muscle such as oxygen transport e.g. *myoglobin* [21, 22]. Suppression of MEF2C has been reported in several skeletal muscle disorders e.g. muscular dystrophy and microgravity induced atrophy [23, 24]. Moreover, *Mef2c* has been shown to be downregulated in different wasting conditions including cancer cachexia [25].

In this study, the Colon 26 (C26) carcinoma mouse model of cachexia was used for the molecular and ultrastructural analyses in wasted skeletal muscle [3, 5, 26]. IL-6 is predominantly responsible for skeletal muscle changes and aberrant metabolism in this model [6, 27]. In this study we demonstrate a novel association between dysregulated MEF2C gene expression and prominent ultrastructural changes in muscle structural integrity and mitochondria in cancer cachexia.

## RESULTS

### Features of the C26 carcinoma model

**Figure 1:**
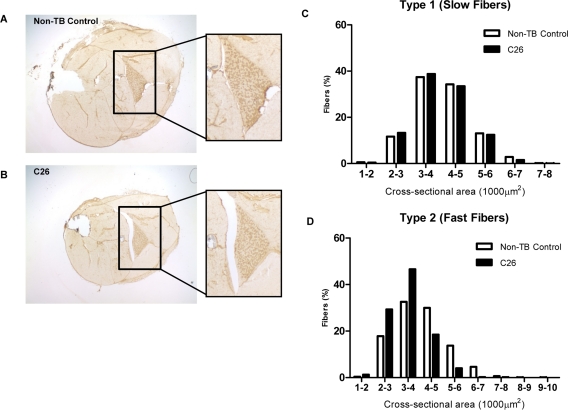
Features of the C26 model of cachexia Frozen sections of lower hindlimbs of (**A**) non-tumor-bearing (non-TB) control and (**B**) C26-bearing mice were immunohistochemically stained for MHC type I protein. Positive myofibers stained a relative dark brown compared to myofibers negative for the protein of interest. (**C-D**) The whole soleus muscle was subjected to myofiber cross-sectional area analysis to compare the proportion of myofiber type 1 (positively stained) and type 2 (negatively stained) in tumor-bearing mice versus non-TB controls. A more prominent reduction of bigger myofibers and a corresponding increase of smaller myofibers was seen in type 2 myofibers (n = 3).

Reduced body and muscle weight associated with elevated plasma IL-6 levels was observed in C26-bearing mice at the experimental endpoint for weight loss in this study (Table 1). The weight loss was associated with myofiber atrophy with a more prominent change in type 2 myofiber cross-sectional area and agreed with previous study which showed the change in diameter ([30]; Figure 1).

### Increased expression of IL-6 signaling molecules

**Figure 2:**
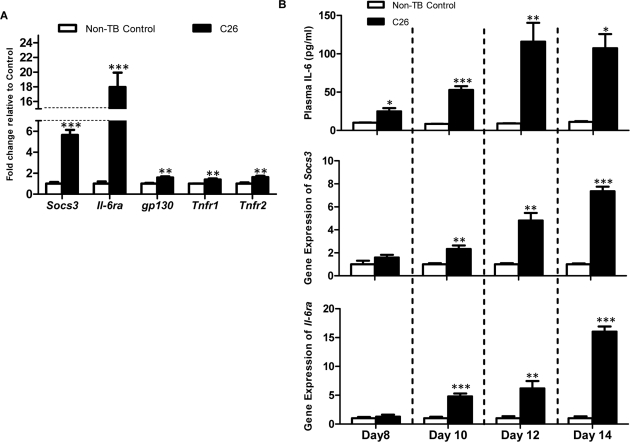
mRNA expression of signaling molecules of the IL-6 signal transduction cascade (**A**) Gene expression of *Socs3*, *Il6ra*, *gp130*, *Tnfr1* and *Tnfr2* were assessed as an indicator of inflammatory signaling (n = 4). Expression of all genes was increased at the endpoint in C26-bearing mice. (**B**) Longitudinal experiments demonstrated the rise of plasma IL-6 preceded the significant increase of *Socs3* and *Il-6ra* at the transcript level. Data are presented as arithmetic means ± SEM. *p < 0.05, **p < 0.01, ***p < 0.001 compared to non-TB controls (n = 4 expect the non-TB groups on day 8 (n = 3) and day 14 (n = 2)).

Expression of the *Socs3* gene, a direct target of activated STAT3 downstream of the IL-6 receptor, has been shown to correlate negatively with skeletal muscle protein content after IL-6 infusion [15]. A significant elevation of *Socs3* transcription was specific to C26-bearing mice at the endpoint (5.64-fold; p < 0.001; Figure 2A). Similarly, gene expression of the IL-6 receptor subunit *Il-6ra* was upregulated in C26-bearing mice (18.0-fold; p < 0.001). A 1.58-fold increase was also seen for its partner, *gp130* in C26-bearing mice compared to the non-TB controls (p < 0.01). Receptors for TNF-α also demonstrated a significant increase in C26-bearing animals (*Tnfr1*: 1.39-fold, *Tnfr2*: 1.62-fold; p < 0.01). Longitudinal studies demonstrated that the increase in circulating IL-6 preceded a significant elevation of the gene expression of *Socs3* and *Il-6ra* (Figure 2B).

### Ultrastructural changes in sarcomeric integrity in muscle

**Figure 3:**
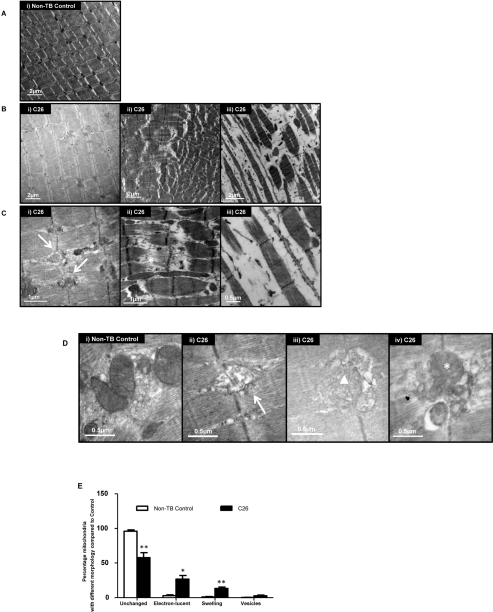
Ultrastructural changes in GAS (**A**) Representative electron micrograph of muscle from non-tumor-bearing (non-TB) control mice. Magnification: × 8000. (**B**) Representative electron micrographs of muscle from C26-bearing mice. Magnification: × 8000 (i & ii), × 10000 (iii). (**C**) A higher magnification of muscle from C26-bearing mice. Vesicle-like structures (arrow); apparent tearing of myofiber (asterisk). Magnification: × 25000 (i & ii), × 30000 (iii). (**D**) Representative electron micrographs of muscle highlighting the morphologies of mitochondria. Electron-lucent areas (arrow); swelling (triangle); vesicle-like structures (asterisk). Magnification: × 40000 (i, ii & iv), × 30000 (iii). (**E**) Percentage of mitochondria with different morphologies in C26-bearing and non-TB mice. Data are presented as arithmetic means ± SEM. *p < 0.05, **p < 0.01 (n = 3). A reduced proportion of normal mitochondria and increased percentage of abnormal mitochondria with various changes were seen in C26-bearing mice compared to non-TB animals.

Analysis of muscle from control mice using electron microscopy showed the parallel register of myofibrils consisting of sarcomeres in series (Figure 3A). The sarcomere is the functional and structural unit for contraction and is demarcated by two Z-lines. Sections from cachectic muscle harvested at the endpoint exhibited altered ultrastructure to varying degrees in different myocytes (Figure 3B-C). Vesicle-like structures were apparent at the Z-lines, and invaded into surrounding myofibrils (Figure 3C (i); arrow) [31]. Sarcomere disintegration was also evident. Extreme changes were seen where the sarcomeres appeared ‘torn’ at the M-line (Figure 3C (ii); asterisk). In other fields of view, sarcomeres were absent with increased interstitial spaces between myofibrils (Figure 3C (iii)).

### Ultrastructural changes in mitochondria in muscle

The normal appearance of mitochondria with densely packed cristae and homogenous matrix was diminished in muscles of C26-bearing mice (Figure 3D). Mitochondrial changes such as the presence of electron-lucent areas (Figure 3D (ii); arrow) and swelling (Figure 3D (iii); triangle) were seen. Some mitochondria were associated with multiple vesicle-like structures which may represent autophagic bodies in the myocyte (Figure 3D (iv); asterisk). A reduced proportion of normal mitochondria in C26-bearing mice (57.5%) compared to non-TB mice (96.1%; p < 0.01; Figure 3E) was apparent. In contrast, the proportion of mitochondria with locally electron-lucent matrix increased in the C26 cachectic group (26.7%) compared to the control group (2.76%; p < 0.05). Swelling and/or fragmentation features of cristae were more apparent in mitochondria of C26-bearing mice (13.2%) compared to the controls (0.95%; p < 0.01). More mitochondria were associated with vesicles in the C26 mice (2.60%) compared to controls (0.23%), although statistical significance was not attained.

### Altered expression of MEF2C and target genes in structural maintenance and energy homeostasis

**Figure 4:**
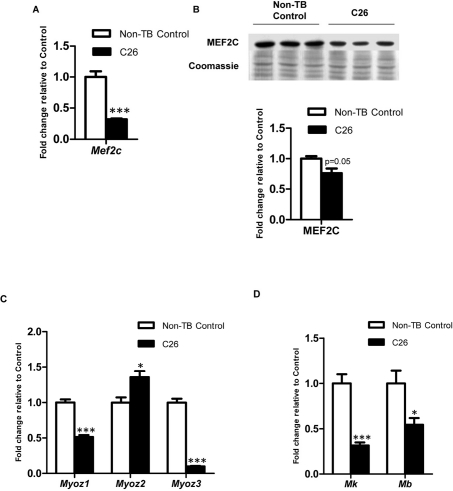
Expression of MEF2C and gene targets governing muscle structural integrity and energy homeostasis Expression of MEF2C at (**A**) gene (n = 4) and (**B**) protein levels (n = 3). Expression of MEF2C target genes which govern (**C**) muscle structural intergrity and (**D**) energy homeostasis. Data are presented as arithmetic means ± SEM. *p < 0.05, ***p < 0.001 compared to non-tumor-bearing (non-TB) controls (n = 4). Expression of MEF2C was downregulated at both mRNA and protein levels. Altered mRNA expression was also seen in *myozenins* (*myoz*), *myokinase* (*Mk*) and *myoglobin* (*Mb*) which indicated disrupted muscle structure integrity and energy homeostasis in skeletal muscle during cancer cachexia.

*Mef2c* mRNA was significantly suppressed in C26-bearing mice compared to non-TB controls (3.13-fold reduction; p < 0.001; Figure 4A). The change was also reflected at the protein level with a 1.32-fold reduction (p = 0.05; Figure 4B). Sarcomeric subtype-specific gene expression changes were observed for the gene encoding a Z-line protein, *Myozenin* (*Myoz*). *Myoz1* was suppressed significantly in cachectic muscle (1.94-fold reduction; Figure 4C) while *Myoz2* in contrast was significantly elevated in C26-bearing mice (1.36-fold). Changes in *Myoz3* expression were similar to *Myoz1* but the extent of downregulation was greater in the cachectic group (10.1-fold reduction). *Myokinase* (*Mk*) was significantly downregulated in C26-bearing mice16-fold reduction; Figure 4D). *Myoglobin* (*Mb*) was also significantly suppressed in C26-bearing mice compared to the control group (1.84-fold reduction).

### Altered expression of calcineurin

**Figure 5:**
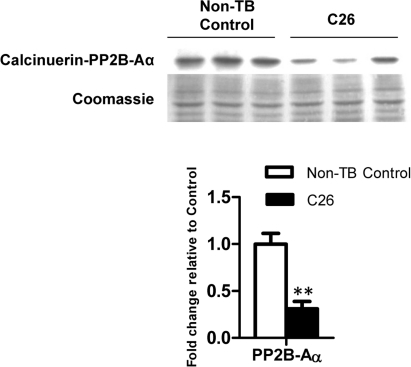
Protein expression of calcineurin The catalytic subunit of calcineurin was reduced at the protein level at endpoint cachexia. Data are presented as arithmetic means ± SEM. **p < 0.01 compared to non-tumor-bearing (non-TB) controls (n = 3).

One of the important upstream regulators of MEF2C is calcineurin, which is a calcium/camodulin-activated protein phosphatase [32]. A 3.21-fold reduction of the calcineurin catalytic subunit PP2B-Aα was observed in C26-bearing mice compared to the non-TB controls (p < 0.01; Figure 5).

## DISCUSSION

The data presented in this study provide further insights into the molecular basis of muscle wasting due to cancer cachexia and highlight the potential role of MEF2C in the development of cachexia. A novel observation of dysregulated myogenic gene expression and associated ultrastructural mitochondrial and sarcomeric changes in the C26 mouse model of can cachexia suggests compromised energy homeostasis and sarcomeric integrity in skeletal muscle during cancer cachexia. These effects during the course of disease progression may be related to altered muscle properties such as weakness and fatigue.

Significant changes in gene expression of relevant molecules in the context of increased circulating IL-6 were seen in muscle from C26-bearing mice. Altered *Socs3* mRNA levels have previously indicated perturbations in IL-6 signaling [15]. In this study, elevated *Socs3* and *Il-6ra* likely resulted from the action of circulating IL-6 on skeletal muscle during cancer cachexia, as the rise in gene expression appeared to follow elevation of circulating IL-6 (Figure 2B). The extent of *Il-6ra* elevation in cachectic muscles compared to controls was greater than that of *Socs3*; suggesting that an increase in IL-6 receptor gene expression in cachectic muscles could further propagate IL-6 signaling. Moreover, the IL-6/IL-6RA complex has been shown to be internalized and degraded, therefore production of IL-6 receptor would be essential to sustain IL-6 signaling [33]. A soluble form of IL-6RA may be generated by proteolytic cleavage or alternative splicing [34]. Therefore, upregulated gene expression of *Il-6ra* in muscle may increase production of soluble IL-6RA and its subsequent release into the circulation, impacting on tissues other than the muscle. This may explain the unexpected fold-changes in the receptor compared to the downstream target, *Socs3*. Gp130, despite being a heterodimer partner of IL-6RA, is ubiquitously expressed and non-specific for IL-6; hence may explain for the lesser degree of elevation. Receptors for TNF-α, also showed a much lower elevation, indicating that TNF-α signaling may not be as crucial as IL-6 signaling in this particular model of cachexia [11].

We have identified novel ultrastructural features in gastrocnemius muscles of C26-bearing mice with myocytes demonstrated to have varying degrees of ultrastructural changes. The data suggests that disruption of myofibrils may have begun at the sarcomeric Z-lines (Figure 3A-C). In areas with a complete loss of sarcomeres, a reduced contractility would be assumed as the functional unit, i.e. sarcomeres were ‘torn’ or had undergone breakdown. The observed ultrastructural changes in the skeletal muscle of C26-bearing mice are novel and may partly explain the asthenia and fatigue which patients with cancer cachexia experience. We propose that the observed ultrastructural changes are linked with altered gene expression of *Mef2c* and its target genes. The importance of MEF2C in regulating postnatal sarcomeric integrity and muscle function has been previously suggested [20]. Gene expression of *Myoz1* and *Myoz3* were downregulated along with *Mef2c* (Figure 4). Expression of *Myoz* has been shown to be suppressed with *Mef2c* muscle-specific deletion, hence likely to be transcriptionally regulated by MEF2C [20]. Myozenins have been reported to interact with a number of Z-line-localised proteins including α-actinin and telethonin in addition to modulating signal transduction via calcineurin [35-37]. Therefore, their reduced expression may compromise muscle integrity, especially at the Z-line. However, the disconnection between *Mef2c* and *Myoz2* gene expression suggests negative regulation by MEF2C in the presence or absence of possibly another regulatory molecule or an alternative, as yet unknown regulatory mechanism independent of MEF2C. For instance NF-κB has previously been shown to be a mediator of *Myoz2* transcription but not *Myoz1* in C2C12 myogenic cells [38]. NF-κB has been associated with skeletal muscle wasting which regulates protein degradation and the ubiquitin-proteasome pathway [39]. Therefore, it is not unreasonable to speculate that different isoforms of the *Myoz* family could be affected via different pathways during cachexia progression.

Morphological alterations were evident in the mitochondria of cachectic muscle (Figure 3D). Semi-quantification of mitochondrial morphological changes provided comprehensive information on the disruption of energy homeostasis in skeletal muscle. These changes included electron-lucent areas which corresponded to a loss of cristae in these mitochondria; suggesting defective oxidative phosphorylation to generate ATP. In addition, swollen mitochondria, which are reported to be associated with cellular ATP depletion, were present [29]. Vesicle-like structures, likely to be autophagic bodies were also observed in some mitochondria which suggested eventual mitochondrial loss via autophagy in cachectic muscle [31]. These observations may reflect the reduced gene expression of *Mk* and *Mb*, which serve to replenish ATP from ADP in the cytosol and transport oxygen respectively. Importantly, myokinase-deficient skeletal muscle has been associated with reduced energy efficiency with contractile performance maintained via compensatory mechanisms e.g. via creatine kinase [40]. This however does not appear to be operational in muscle loss due to cancer cachexia (unpublished data). Moreover, *Mb* has been known as a MEF2 transcriptional target; further establishing the role of MEF2C during cancer cachexia in compromising not only structural integrity but also energy metabolism [41].

**Figure 6:**
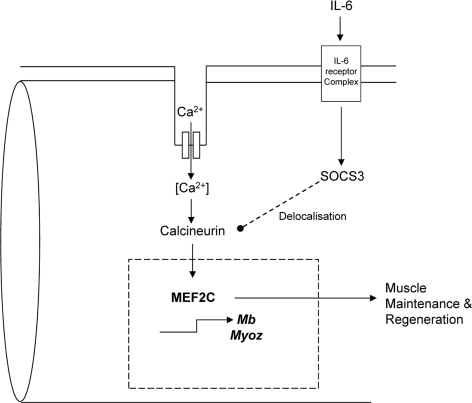
A proposed model of IL-6 dependent loss of sarcomeric integrity a nd function involving MEF2C Expression and activity of MEF2C are regulated by calcineurin. Chronic activation of IL-6 signaling in skeletal muscle results in an upregulated expression of SOCS3. SOCS3 has been shown to delocalize calcineurin from its usual Z-line position to the periphery of a myofiber. This might affect its function and hence impact on the downstream targets like MEF2C. Since MEF2C is a key regulator of many myogenic and energy homeostatic molecules, any perturbation in its activity could greatly affect muscle performance and integrity. Such alterations may accelerate muscle breakdown and disintegration of the tissue contributing to fatigue and weakness during cancer cachexia.

The reduced expression of the catalytic subunit of calcineurin suggested a pathway for the reduced *Mef2c* expression seen (Figure 5). Interestingly, calcineurin has been shown to be delocalized by SOCS3 in skeletal muscle from the usual site along the Z-line in a SOCS3 transgenic mouse model [42]. Therefore, we propose a novel link between elevated plasma IL-6 and suppressed *Mef2c* transcription via calcineurin (Figure 6). This raises a new perspective for muscle wasting in cancer cachexia whereby degradation could be accelerated through disruption of sarcomeric integrity. Elucidating the mechanism of such disruption would provide new molecular targets for interventions in controlling muscle loss. Moreover, it would also be beneficial in the context of aging since mitochondrial dysfunction, reduced insulin sensitivity and declined muscle performance are often presented in aged people [43]. It should also be noted that MEF2C is a transcriptional regulator of Glucose transporter type (GLUT) 4, the main glucose transporter in skeletal muscle, hence the disrupted MEF2C signaling could impact on insulin sensitivity and should be addressed in future studies [44]. Therefore it appeared that MEF2C is a promising target for intervention strategies to reverse or stabilize muscle loss in cachexia. Future research into molecular mechanisms for muscle loss and intervention strategies to correct the effects of dysregulated MEF2C would greatly enhance our understanding of cancer cachexia progression and ultimately benefit cancer patient quality of life by improving muscle strength.

## MATERIALS AND METHODS

### C26 mouse model of cachexia

C26 carcinoma cells (Amgen, Thousand Oaks, California) were maintained in RPMI/Penicillin Streptomycin (PS) in a humidified atmosphere with 5% CO_2_ at 37°C. Male Balb/c-DBA hybrid mice (10-11 weeks old) were obtained from the Animal Resources Centre (Perth, WA, Australia) and housed at the Molecular Physiology Unit (ANZAC Research Institute, Concord, NSW, Australia). Mice were maintained at a constant temperature (22°C) on a 12 hr light: 12 hr dark cycle. Standard rodent chow and water *ad libitum* were freely accessible. All animal work prior to culling was conducted under approval (Sydney South West Area Health Service Animal Welfare Committee; protocol number 2007/006). For tumor inoculation, 1 × 10^6^ C26 cells in 100 μl RPMI/PS were subcutaneously inoculated in the upper right flank of the mice. Non-tumor-bearing (Non-TB) control mice were injected with 100 μl RPMI/PS.

### Plasma IL-6 measurements

Mice were anesthetised at the endpoint (Day 8, 10, 12 or 14) by intraperitonial injection of ketamine (100 mg/kg) and xylazine (50 mg/kg). Blood was then collected by cardiac puncture and spun at 5000 × g for 5mins for plasma isolation. Quantikine® Mouse IL-6 Immunoassay kit (R&D Systems, Minneapolis, MN, USA) was used to determine plasma IL-6 concentration following the manufacturer's protocol.

### Tissue weights and tissue collection

Mice were sacrificed by cervical dislocation at 2 pm. For the longitudinal study, mice were culled on day 8, 10, 12 or 14 post-tumor inoculation. For single time-point experiments, endpoint was selected so that percentage weight loss in C26-bearing mice ranged from 13-18%, which was within the ethnical limit of 20%. Mice were culled on day 14 and 16 post-tumor inoculation to account for the inter-individual variability of weight loss. In preparation for histological studies, lower hindlimbs were embedded in Tissue-Tek® (Pro-Sci Tech,Thuringowa, QLD, Australia) and frozen over isopentane cooled on liquid nitrogen. Individual muscles were isolated, weighed and subsequently snap-frozen in liquid nitrogen and stored at −80°C.

### Immunohistological studies

Frozen lower hindlimbs were sectioned at 7-10μm on a Leica CM1900 cryostat. Immunohistochemistry for myosin heavy chain (MHC) isoforms representative of different myofiber types were performed as previously described [28]. Each section was digitally photographed with 1.25 × and 10 × objectives for cross-section of the whole lower hindlimb and the soleus respectively (Zeiss Axio Imager A1microscope with a Spot Insight Firewire camera Model 11.3 and Spot Version 4.0.2 software; Diagnostic Instrument Inc., Sterling Heights, MI, USA). Myofiber counts and measurements were then performed on the soleus muscle.

### Electron microscopy

Ultrathin sections (70 nm) were cut from fixed gastrocnemius muscles and stained with 2% uranyl acetate (aqueous) and 2% lead citrate and examined under a Jeol1400 transmission electron microscope (Electron Microscopic Unit, UNSW, NSW, Australia) at a voltage of 100 kV. Semi-quantitative scoring was performed according to [29] with modifications. Electron micrographs taken at a magnification of × 25000 were used. Multiple images were randomly taken from one grid. Mitochondria were categorized into four groups according to their morphology: unchanged (group 1), electron-lucent matrix (group 2), swelling/ fragmentation of cristae (group 3), association with vesicle-like structures (group 4).

### Gene expression studies

Total RNA was extracted from gastrocneumius muscle using TRIzol® reagent (Invitrogen, Carlsbad, CA, USA). RNA (0.5 μg) was used in a first-strand reaction with oligo dT primers (Invitrogen) and Superscript® reverse transcriptase (Invitrogen). Real-time quantitative polymerase chain reaction (PCR) was performed using specific primers (Sigma, Castle Hill, NSW, Australia; Supplementary Table 1) on a Corbett RG 6000 rotor machine (Corbett Research, Mortlake, VIC, Australia). Each standard reaction contained 5 μl sample cDNA (1:100 dilution); 12 μl SYBR® Green (Invitrogen); 6 μl RNase free water and 1 μl each of forward and reverse primers (300nM). *TATA box-binding protein* (*Tbp*) and *ribosomal protein, large, P0* (*36B4*) were used as the housekeeping genes to obtain normalization factors from geNorm Version 3.5 (Ghent University Hospital, Center for Medical Genetics, Ghent, Belgium). Normalization for candidate gene expression was corrected against housekeeping gene expression; each candidate gene was divided by its normalization factor for each mouse muscle sample.

### Protein expression studies

Muscle extracts were prepared by homogenising gastrocnemius muscle in ice-cold RIPA buffer (Cell Signaling Technology, Danvers, MA, USA) supplemented with protease inhibitor (Roche Diagnostics, Sydney, Australia), incubated on ice for 1 hr. Supernatant lysates were collected following centrifugation at 14000 × g for 10 min at 4°C. Twenty micrograms of extract were subjected to 10% SDS-polyacrylamide gel electrophoresis and transferred to PVDF membranes (Bio-Rad Laboratories, Gladesville, NSW, Australia). Blocking was performed in 1% polyvinylpyrrolidine-40 (PVP-40) – 1 × TBST (20 mM Trizma Base [pH7.5], 135 mM NaCl, 0.1% Tween 20) followed by 3% skim milk powder-1% PVP-40 – 1 × TBST. Primary and secondary antibodies were diluted in 0.5% PVP-40 – 1 × TBST and 1% PVP-40 – 1 × TBST for an overnight and 1 hr incubation at 4°C and room temperature respectively. Washes were performed in 1 × TBST for 5 to 10 min and repeated five times. Specific proteins were visualized by SuperSignal West Femto Substrate (Thermo Scientific Inc., Scoresby, VIC, Australia). Antibodies to MEF2C and PP2B-A were obtained from Santa Cruz Biotechnology (California, USA).

### Statistical analysis

Data are presented as arithmetic means ± SEM. Two-tailed unpaired t-tests were performed to compare C26-bearing and non-TB control groups. A P value of <0.05 was considered as significant. GraphPad Prism 5.03 (GraphPad Software, San Diego, CA) was used for all data analysis and preparation of graphs.

## SUPPLEMENTARY TABLE

Supplementary Table 1 Primers used for quantitative real-time PCR
