# Supplementary figures and images for: Disruption of MEF2C signaling and loss of sarcomeric and mitochondrial integrity in cancer-induced skeletal muscle wasting
Source: Aging (Albany NY). 2012 Feb 21;4(2):133–43. doi: 10.18632/aging.100436 (PMC3314175; doi:10.18632/aging.100436)

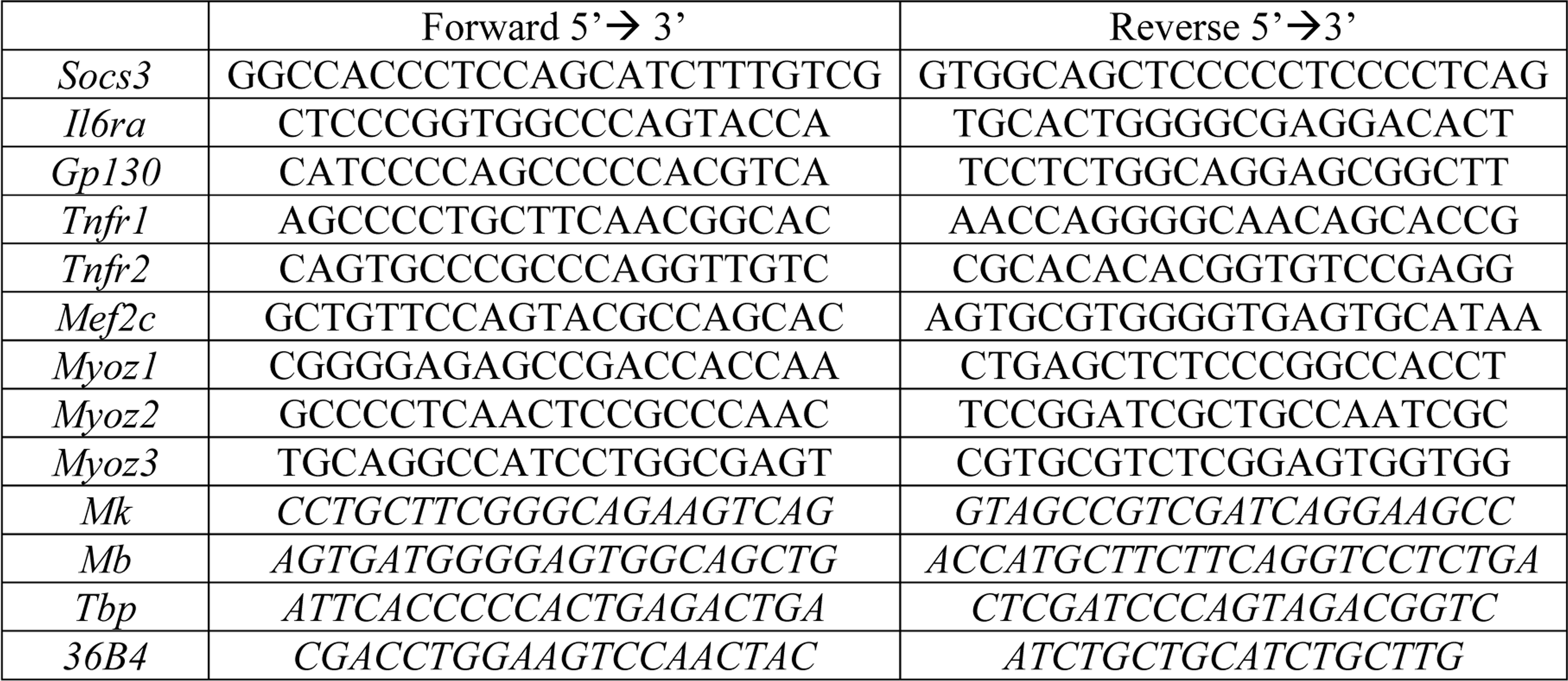

Supplement: Supplementary Table 1 [file aging-04-133-s001.tif]
